# Synthesis, Molecular Structure and Cytotoxicity of Molecular Materials Based on Water Soluble Half-Sandwich Rh(III) and Ir(III) Tetranuclear Metalla-Cycles

**DOI:** 10.3390/ma6115352

**Published:** 2013-11-20

**Authors:** Gajendra Gupta, Benjamin S. Murray, Paul J. Dyson, Bruno Therrien

**Affiliations:** 1 Institute of Chemistry, University of Neuchatel, Avenue de Bellevaux 51, Neuchatel CH-2000, Switzerland; E-Mail: gajendra.gupta@unine.ch; 2 Institute of Chemical Sciences and Engineering, Ecole Polytechnique Fédérale de Lausanne (EPFL), Lausanne CH-1015, Switzerland; E-Mails: benjamin.murray@epfl.ch (B.S.M.); paul.dyson@epfl.ch (P.J.D.)

**Keywords:** metalla-cycles, half-sandwich complexes, bioorganometallic chemistry, metal-based drugs, metalla-rectangles, anticancer activity

## Abstract

The neutral dinuclear complexes [(η^5^-C_5_Me_5_)_2_Rh_2_(μ-dhnq)Cl_2_] (**1**) and [(η^5^-C_5_Me_5_)_2_Ir_2_(μ-dhnq)Cl_2_] (**2**) (dhnqH_2_ = 5,8-dihydroxy-1,4-naphthoquinone) were obtained from the reaction of [(η^5^-C_5_Me_5_)M(μ-Cl)Cl]_2_ (M = Rh, Ir) with dhnqH_2_ in the presence of CH_3_COONa. Treatment of **1** or **2** in methanol with linear ditopic ligands L (L = pyrazine, 4,4′-bipyridine or 1,2-bis(4-pyridyl)ethylene), in the presence of AgCF_3_SO_3_, affords the corresponding tetranuclear metalla-rectangles [(η^5^-C_5_Me_5_)_4_M_4_(μ-dhnq)_2_(μ-L)_2_]^4+^ (L = pyrazine, M = Rh, **3**; M = Ir, **4**; L = 4,4′-bipyridine, M = Rh, **5**; M = Ir, **6**; L = 1,2-bis(4-pyridyl)ethylene, M = Rh, **7**; M = Ir, **8**). All complexes were isolated as their triflate salts and were fully characterized by infrared, ^1^H and ^13^C NMR spectroscopy, and some representative complexes by single-crystal X-ray structure analysis. The X-ray structures of **3**, **5** and **6** confirm the formation of the tetranuclear metalla-cycles, and suggest that complexes **5** and **6** possess a cavity of sufficient size to encapsulate small guest molecules. In addition, the antiproliferative activity of the metalla-cycles **3**–**8** was evaluated against the human ovarian A2780 (cisplatin sensitive) and A2780cisR (cisplatin resistant) cancer cell lines and on non-tumorigenic human embryonic kidney HEK293 cells. All cationic tetranuclear metalla-rectangles were found to be highly cytotoxic, with IC_50_ values in the low micromolar range.

## 1. Introduction

The biological application of coordination-driven arene ruthenium metalla-materials is a flourishing area of research [1,2,3,4,5,6]. The tetranuclear assemblies have been found to possess good anticancer activity [7,8,9,10,11,12], to strongly interact with DNA [13,14], and to efficiently detect biologically relevant analytes [15,16,17]. Their DNA binding can occur through non-covalent interactions, novel modes of action also observed for di- and trinuclear organometallics [18,19,20], although fragmentation inside cells followed by coordination of the metal ion to DNA and/or other biomolecules cannot be excluded. Following the promising studies of arene ruthenium metalla-materials, a series of cationic arene osmium metalla-rectangles were recently reported [21]. These arene osmium derivatives display comparable cytotoxicity to the ruthenium-based analogues, suggesting that the biological applications of arene ruthenium metalla-rectangles can be extended to other transition metals.

**Figure 1:**
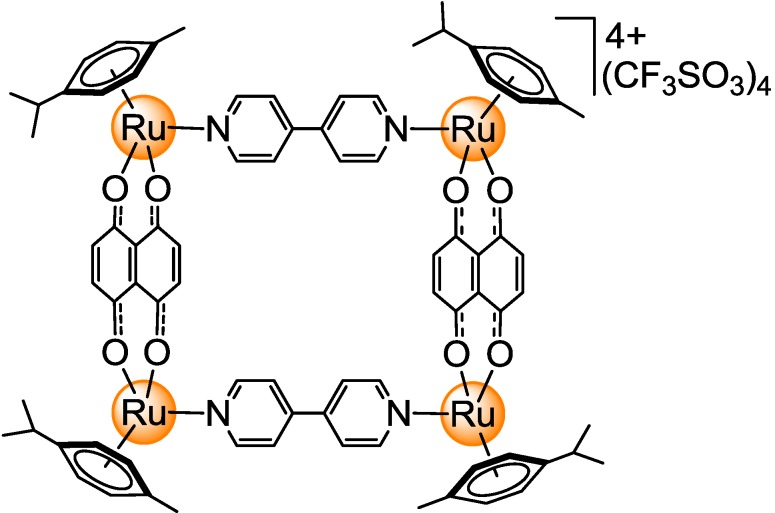
Molecular structure of [(η^6^-*p*-Pr^i^C_6_H_4_Me)_4_Ru_4_(μ-dhnq)_2_(μ-4,4′-bipyridine)_2_](CF_3_SO_3_)_4_.

It is well known that the chemistry of arene ruthenium and arene osmium complexes is similar to that of half-sandwich rhodium and iridium complexes [22,23,24,25] and that several organometallic compounds of these metals with promising anticancer activity have been reported [26,27,28,29]. Moreover, several pentamethylcyclopentadienyl rhodium and iridium metalla-rectangles have been synthesized and structurally characterized by the group of Jin [30,31,32]. These metalla-rectangles show comparable structural and physico-chemical properties to the arene ruthenium analogues. However, the biological activity of these tetranuclear assemblies has not yet been explored. Therefore, to evaluate the anticancer properties of pentamethylcyclopentadienyl rhodium and iridium metalla-rectangles, a series of cationic tetranuclear complexes incorporating 5,8-dioxido-1,4-naphthoquinonato (dhnq) bridges and ditopic N-ligands [pyrazine, 4,4′-bipyridine and 1,2-bis(4-pyridyl)ethylene] has been prepared and their antiproliferative activity evaluated* in vitro* on human ovarian cancer cell lines (A2780 and A2780cisR) and on non-tumorigenic cells (HEK293). The anticancer activity of these pentamethylcyclopentadienyl rhodium and iridium metalla-rectangles was compared to that of [(η^6^-*p*-Pr^i^C_6_H_4_Me)_4_Ru_4_(μ-dhnq)_2_(μ-4,4′-bipyridine)_2_](CF_3_SO_3_)_4_ (Figure 1) [9].

## 2. Results and Discussion

**Scheme 1:**
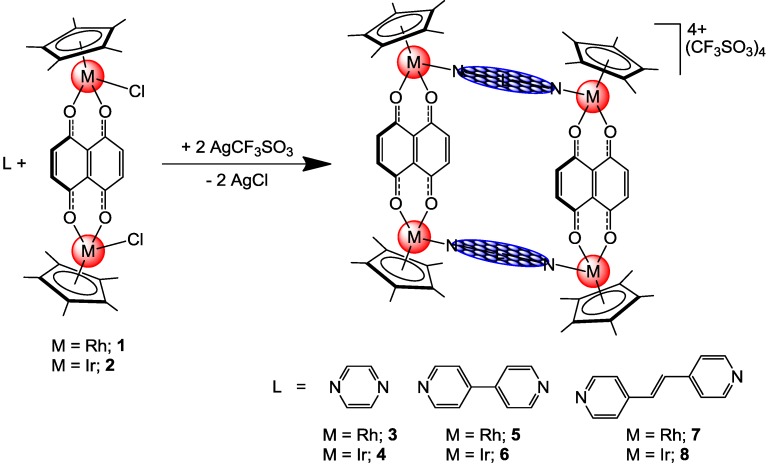
Synthesis of tetranuclear metalla-rectangles **3**–**8** from **1** and **2**.

The reaction of the dinuclear pentamethylcyclopentadienyl complexes [(η^5^-C_5_Me_5_)M(μ-Cl)Cl]_2_ (M = Rh and Ir) with dhnqH_2_ (dhnqH_2_ = 5,8-dihydroxy-1,4-naphthoquinone) in methanol in the presence of CH_3_COONa leads to the formation of the neutral complexes [(η^5^-C_5_Me_5_)_2_Rh_2_(μ-dhnq)Cl_2_] (**1**) and [(η^5^-C_5_Me_5_)_2_Ir_2_(μ-dhnq)Cl_2_] (**2**). Addition of AgCF_3_SO_3_ to **1** and **2**, followed by addition of linear ditopic ligands L [L = pyrazine, 4,4′-bipyridine or 1,2-bis(4-pyridyl)ethylene], affords the cationic tetranuclear metalla-rectangles of the general formula [(η^5^-C_5_Me_5_)_4_M_4_(μ-dhnq)_2_(μ-L)_2_]^4+^ (L = pyrazine, M = Rh, **3**; M = Ir, **4**; L = 4,4′-bipyridine, M = Rh, **5**; M = Ir, **6**; L = 1,2-bis(4-pyridyl)ethylene, M = Rh, **7**; M = Ir, **8**), see Scheme 1. The rectangular cations **3**–**8** are isolated as triflate salts. All complexes are non-hygroscopic and stable in air and have been fully characterized by analytical and spectroscopic techniques. The compounds are soluble in polar solvents and are insoluble in non-polar solvents. In addition, all the metalla-rectangles are soluble and stable in water and DMSO (dimethyl sulfoxide). Interestingly, the metalla-rectangles are stable for weeks in water, while in DMSO, new species only start to appear after 3–4 h in solution [determined by nuclear magnetic resonance (NMR)].

**Figure 2:**
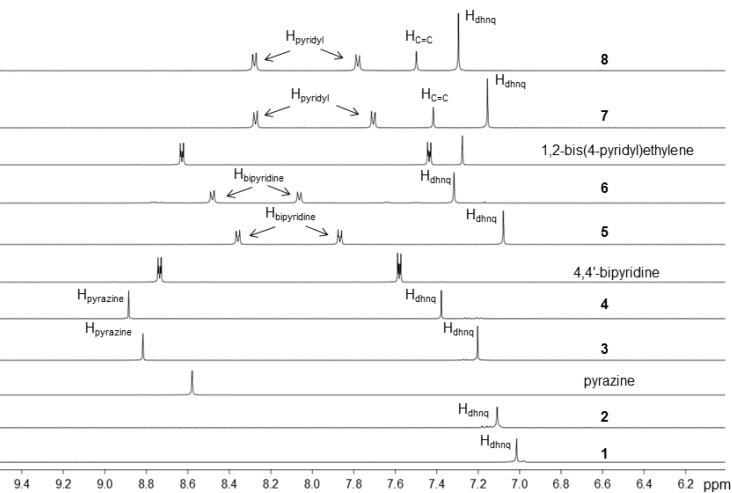
^1^H NMR spectra (aromatic region) of free pyrazine, 4,4′-bipyridine and 1,2-bis(4-pyridyl)ethylene, as well as the dinuclear complexes **1**–**2** and the metalla-rectangles **3**–**8** in CD_2_Cl_2_ (25 °C).

The ^1^H NMR spectra of the complexes were recorded in CD_2_Cl_2_ at 25 °C and the chemical shifts of the different protons are listed in the experimental section. The ^1^H NMR spectra of **1**–**8** contain a singlet resonance between 7.0 and 7.4 ppm that can be attributed to the protons of the dhnq bridges. Upon formation of the metalla-rectangles, the signals associated to the dhnq protons are shifted downfield relative to those of the neutral dinuclear complexes **1** (δ = 7.01 ppm) and **2** (δ = 7.09 ppm), see Figure 2. Similarly, the singlet of the pyrazine ligands is shifted downfield after formation of metalla-rectangles **3** and **4**. In contrast, the proton signals derived from the 4,4′-bipyridine and 1,2-bis(4-pyridyl)ethylene derivatives show a different trend after coordination, the H_α_ of the pyridyl groups are shifted upfield whereas the H_β_ protons are shifted downfield, see Figure 2. The observed chemical shifts are consistent with the formation of the expected metalla-rectangles.

### 2.1. Molecular structures of **3**, **5** and **6**

**Figure 3:**
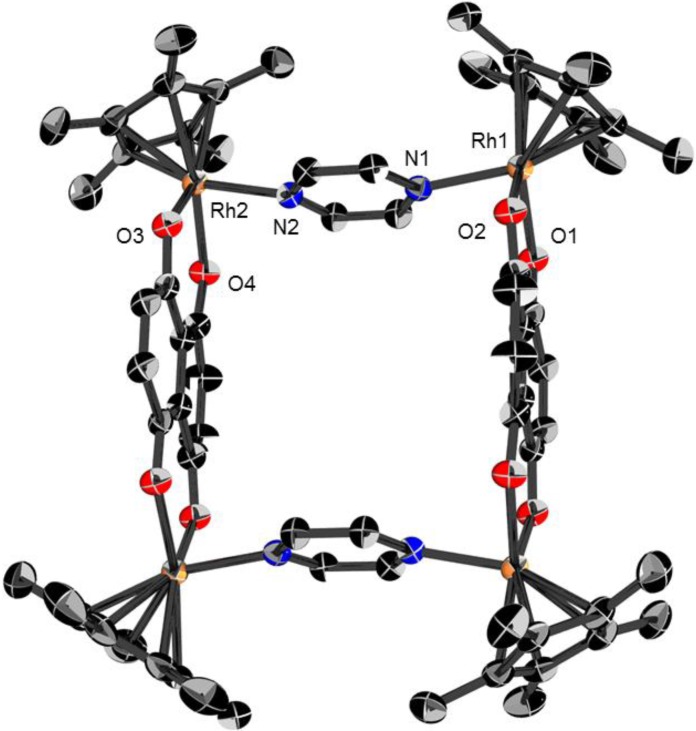
Molecular structure of metalla-rectangle **3** at 50% probability level ellipsoids with hydrogen atoms, dichloromethane molecules and triflate anions omitted for clarity.

**Figure 4:**
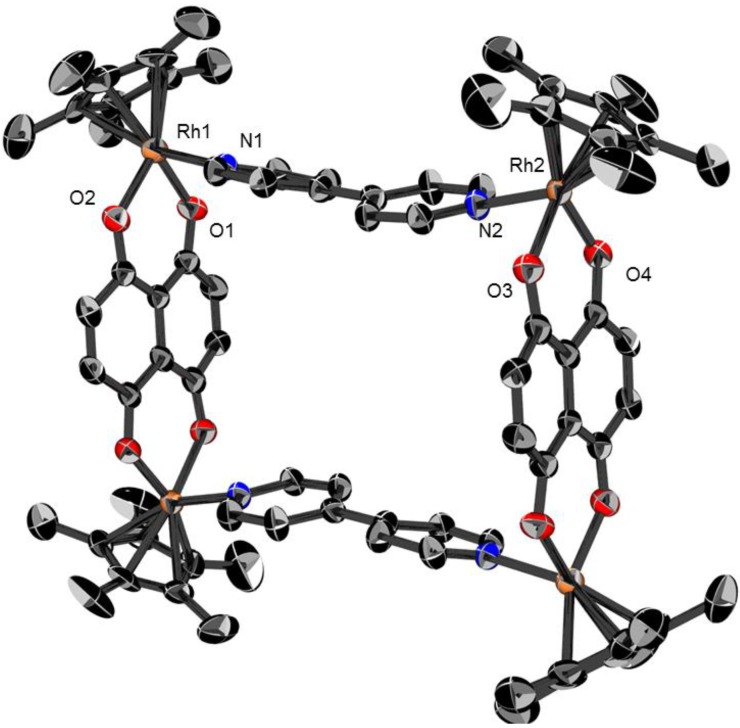
Molecular structure of metalla-rectangle **5** at 50% probability level ellipsoids with hydrogen atoms, dichloromethane molecules and triflate anions omitted for clarity.

**Figure 5:**
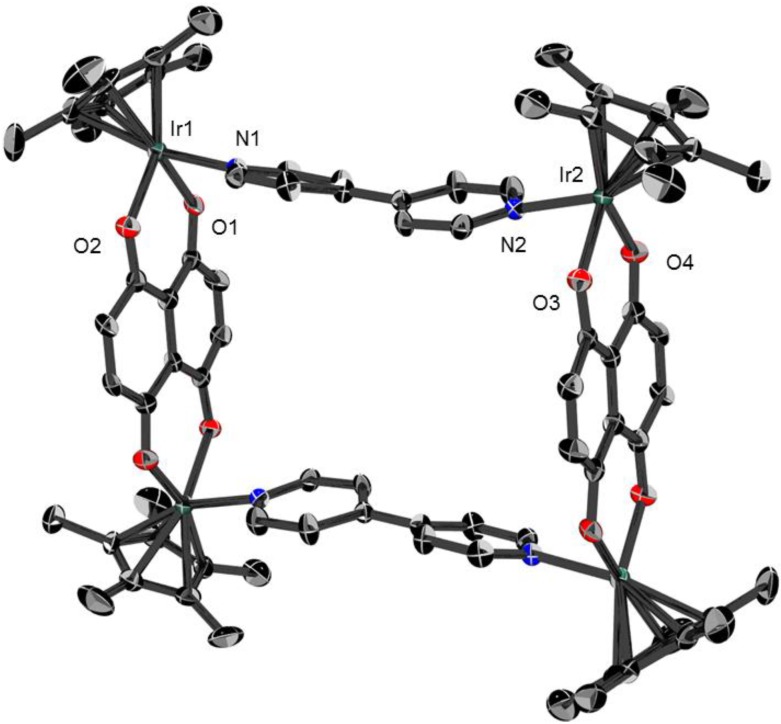
Molecular structure of metalla-rectangle **6** at 50% probability level ellipsoids with hydrogen atoms, solvent molecules and triflate anions omitted for clarity.

Dark green crystals of the metalla-rectangles [**3**](CF_3_SO_3_)_4_, [**5**](CF_3_SO_3_)_4_ and [**6**](CF_3_SO_3_)_4_, suitable for single-crystal X-ray structure analysis were obtained by addition of toluene to a dichloromethane solution of the respective complexes. All metalla-rectangles crystallize with solvent molecules and four triflate anions. The molecular structures of **3**, **5** and **6** are presented in Figure 3, Figure 4 and Figure 5, respectively. Selected bond lengths and angles are listed in Table 1 and the crystallographic details are given in Table 2.

As expected, metalla-rectangle **3** is composed of two dinuclear [(η^5^-C_5_Me_5_)_2_Rh_2_(μ-dhnq)]^2+ ^clips connected by two pyrazine ligands (Figure 3). The Rh-Rh distances are 6.9773(5) Å through the pyrazine units and 8.3831(5) Å through the dhnq bridges. In metalla-rectangles **5** and **6**, the dinuclear [(η^5^-C_5_Me_5_)_2_M_2_(μ-dhnq)]^2+ ^clips are connected with 4,4′-bipyridine units, thus providing a much longer rectangle with metal-metal distances through the 4,4′-bipyridine ligands of 11.106(1) and 11.2437(7) Å for **5** and **6**, respectively. The metal-metal distances observed in these metalla-rectangles are comparable to those found in analogous half-sandwich metalla-assemblies incorporating either pyrazine, 4,4′-bipyridine or dhnq building blocks [33,34,35].

The cavity size of metalla-rectangles **5** and **6** is approximately 8 × 11 Å^2^, while for metalla-rectangle **3** the cavity is 8 × 7 Å^2^ (Table 1). Therefore, considering the size of the hydrophobic cavity and the presence of aromatic rings in the multiple components of the metalla-rectangles, the hydrophobic cavities of the larger metalla-rectangles **5**–**8** can potentially provide a site for the inclusion of small guest molecules. The *p-*cymene ruthenium analogues, [(η^6^-*p*-Pr^i^C_6_H_4_Me)_4_Ru_4_(μ-dhnq)_2_(μ-4,4′-bipyridine)_2_]^4+^ and [(η^6^-*p*-Pr^i^C_6_H_4_Me)_4_Ru_4_(μ-dhnq)_2_(μ-1,2-bis(4-pyridyl)ethylene)_2_]^4+^, have been shown to encapsulate pyrene and other planar aromatic molecules in solution [36,37].

**Table 1:** Selected bond lengths and angles for [**3**](CF_3_SO_3_)_4_·4CH_2_Cl_2_; [**5**](CF_3_SO_3_)_4_·2CH_2_Cl_2_ and [**6**](CF_3_SO_3_)_4_·solvent.

	[3](CF_3_SO_3_)_4_·4CH_2_Cl_2_	[5](CF_3_SO_3_)_4_·2CH_2_Cl_2_	[6](CF_3_SO_3_)_4_·solvent
**Interatomic distances (Å)**			
M1-O1	2.065(3)	2.030(4)	2.073(5)
M1-O2	2.053(3)	2.028(5)	2.073(5)
M2-O3	2.061(3)	2.034(5)	2.069(5)
M2-O4	2.061(3)	2.035(4)	2.064(5)
M1-N1	2.150(3)	2.099(5)	2.116(6)
M2-N2	2.129(3)	2.094(5)	2.116(6)
M1-M2 (μ-dhnq)	8.3831(5)	8.283(1)	8.4075(6)
M1-M2 (μ-N-ligand)	6.9773(5)	11.106(1)	11.2437(7)
**Angles (°)**			
O1-M1-O2	87.2(1)	87.5(2)	87.4(2)
O3-M2-O4	87.4(1)	87.1(2)	87.3(2)
N1-M1-O1	84.6(1)	86.8(2)	83.1(2)
N1-M1-O2	85.7(1)	84.0(2)	83.5(2)
N2-M2-O3	84.5(1)	85.6(2)	82.9(2)
N2-M2-O4	85.7(1)	84.5(2)	84.8(2)

**Table 2:** Crystallographic and structure refinement parameters for metalla-rectangles [**3**](CF_3_SO_3_)_4_·4CH_2_Cl_2_; [**5**](CF_3_SO_3_)_4_·2CH_2_Cl_2_; and [**6**](CF_3_SO_3_)_4_·solvent.

Parameter	[3](CF_3_SO_3_)_4_·4CH_2_Cl_2_	[5](CF_3_SO_3_)_4_·2CH_2_Cl_2_	[6](CF_3_SO_3_)_4_·solvent
Chemical formula	C_76_H_84_Cl_8_F_12_N_4_O_20_Rh_4_S_4_	C_86_H_88_Cl_4_F_12_N_4_O_20_Rh_4_S_4_	C_84_H_84_F_12_Ir_4_N_4_O_20_S_4_
Formula weight	2424.95	2407.28	2594.59
Crystal system	Monoclinic	Triclinic	Triclinic
Space group	*P* 2_1_/*c* (no. 14)	*P*-1 (no. 2)	*P*-1 (no. 2)
Crystal color and shape	yellow block	green block	grey block
Crystal size	0.22 × 0.18 × 0.17	0.16 × 0.15 × 0.13	0.21 × 0.20 × 0.16
*a* (Å)	12.3640(4)	12.8157(9)	12.5846(6)
*b* (Å)	23.6046(6)	14.8016(10)	15.2071(7)
*c* (Å)	16.8800(6)	15.0871(10)	15.4594(7)
α (°)	–	90.206(5)	89.590(4)
β (°)	104.176(3)	100.106(5)	80.133(4)
γ (°)	–	106.634(5)	73.386(4)
*V* (Å^3^)	4776.4(3)	2695.2(3)	2790.4(2)
*Z*	2	1	1
*T* (K)	173(2)	173(2)	173(2)
*D_c_* (g·cm^−3^)	1.686	1.483	1.544
μ (mm^−1^)	1.080	0.860	4.906
Scan range (°)	1.70 ˂ θ ˂ 29.23	1.69 ˂ θ ˂ 29.30	1.72 ˂ θ ˂ 29.22
Unique reflections	12909	14597	14976
Reflections used [I > 2σ(I)]	9875	9188	10306
*R*_int_	0.0908	0.1407	0.0623
Final R indices [I > 2σ (I)]^*^	0.0587, *w*R_2_ 0.1202	0.0881, *w*R_2_ 0.2094	0.0532, *w*R_2_ 0.1163
R indices (all data)	0.0836, *w*R_2_ 0.1299	0.1412, *w*R_2 _0.2423	0.0915, *w*R_2_ 0.1274
Goodness-of-fit	1.039	1.103	1.001
Max, Min Δρ (e Å^−3^)	1.079, −1.341	1.775, −1.254	1.925, −2.579

^*^ Structures were refined on F_0_^2^: *w*R_2_ = [Σ[*w* (F_0_^2^ − F_c_^2^)^2^]/Σ*w* (F_0_^2^)^2^]^1/2^, where *w*^−1^ = [Σ(F_0_^2^) + (aP)^2^ + bP] and P = [max(F_0_^2^,0) + F_c_^2^]/3

**Figure 6:**
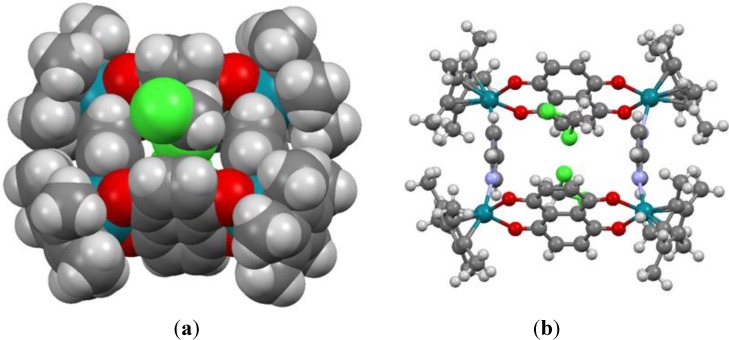
Space-filling (**a**) and ball-and-stick models (**b**) of metalla-rectangle **3** showing the two molecules of dichloromethane sitting on both sides of the cavity.

As previously mentioned, in the crystal packing of **3**, **5** and **6** the solvent molecules and triflate anions are either situated at the periphery of the metalla-rectangles or positioned in the hydrophobic cavity. Despite possessing the smallest cavity, in metalla-rectangle **3** two molecules of dichloromethane sit on both sides of the metalla-cycle, see Figure 6. The shorter Cl∙∙∙Cl separation between the two solvent molecules is 3.265(4) Å. In **5** and **6**, the cavity of the metalla-rectangles is more spacious and accordingly the guest molecules have more freedom, thus they are not as well ordered as in the crystal packing of **3**, and were not refined (see experimental part). Nevertheless, these metalla-materials can act as host compounds with appropriate guest molecules, an interesting property for biological applications [1,2,3,4,5,6].

### 2.2. Antiproliferative Activity

The cytotoxicity of **3**–**8** and a Ru-analogue (Figure 1) was evaluated against human A2780 (cisplatin sensitive) and A2780cisR (cisplatin resistant) ovarian cancer cells, as well as against the non-tumorigenic HEK293 human embryonic kidney cells. The IC_50_ values after 72 h are listed in Table 3. Regarding the neutral dinuclear complexes **1** and **2**, their solubility in water was too low to allow a biological evaluation, and in DMSO the chlorido ligands were exchanged with solvent molecules.

**Table 3:** IC_50_ values of metalla-rectangles **3**–**8** and the Ru-analogue [(η^6^-*p*-Pr^i^C_6_H_4_Me)_4_Ru_4_(μ-dhnq)_2_(μ-4,4′-bipyridine)_2_](CF_3_SO_3_)_4_, toward human ovarian cancer cells A2780 and A2780cisR and healthy cells HEK293 after 72 h exposure.

IC_50_ (μM)			
**Compound**	**A2780**	**A2780cisR**	**HEK293**
cisplatin	1.26 ± 0.17	19.7 ± 3.00	6.55 ± 1.00
**3**	0.06 ± 0.01	0.19 ± 0.01	0.17 ± 0.01
**4**	0.07 ± 0.01	0.25 ± 0.05	0.09 ± 0.02
**5**	0.08 ± 0.01	0.20 ± 0.01	0.09 ± 0.02
**6**	0.13 ± 0.02	0.31 ± 0.04	0.11 ± 0.02
**7**	0.06 ± 0.01	0.18 ± 0.01	0.10 ± 0.01
**8**	0.17 ± 0.01	0.29 ± 0.03	0.10 ± 0.02
Ru-analogue	1.49 ± 0.11	1.94 ± 0.07	0.77 ± 0.03

All the metalla-rectangles **3**–**8** are highly cytotoxic towards the three cell lines tested (0.06–0.31 μM), and are significantly more cytotoxic than the ruthenium analogue and cisplatin in all cases. One observable trend is that all the compounds were less active against the A2780cisR cell line compared to the A2780 cell line, indicative of a certain level of susceptibility of these compounds to the acquired cisplatin resistance mechanisms operating in the former, albeit with notably lower resistance factors (1.3–3.6) relative to cisplatin (15.6). The IC_50_ values of the compounds in the HEK293 cell line are comparable to the corresponding IC_50_ values for the A2780 cell line, with the exception of **3** which is almost three-fold more active in the A2780 cell line (0.06 μM) compared to the HEK293 cell line (0.17 μM), indicative of a moderate level of selectivity. For compounds **3**–**8** the cytotoxicity appears to be independent of the choice of metal (Rh or Ir) or the ditopic nitrogen ligand present, exemplified by the similarity in IC_50_ values determined for **3**–**8** within the three cell lines. In contrast, the activity of the Ru-analogue is significantly lower than its Rh and Ir analogues, **5** and **6**, in all three cell lines, indicating, in this case, the choice of metal (Rh and Ir* versus* Ru) is significant with respect to the level of cytotoxicity observed. Given these results it is tempting to speculate that compounds **3**–**8** exert their cytotoxic activity through a similar mechanism of action. Evidently on switching metal to yield the Ru-analogue the activity of the complex diminishes and is likely related to a fundamental difference in reactivity of this metalla-rectangle* in vitro*.

## 3. Experimental Section

### 3.1. General

The starting materials [(η^5^-C_5_Me_5_)Rh(μ-Cl)Cl]_2_ and [(η^5^-C_5_Me_5_)Ir(μ-Cl)Cl]_2_ were prepared according to published methods [38,39]. All other reagents were purchased and used without further purification. The ^1^H and ^13^C{^1^H} NMR spectra were recorded with a Bruker Avance II 500 or a Bruker Avance II 400 MHz spectrometer (Bruker BioSpin GmbH, Rheinstetten, Germany) using the residual protonated solvent as an internal reference. Infrared spectra were recorded as KBr pellets with a Perkin-Elmer FTIR 1720 X spectrometer (PerkinElmer: Waltham, MA, USA). Microanalyses were carried out by the Mikroelementaranalytisches Laboratorium, ETH Zürich (Switzerland).

### 3.2. Synthesis of [(η^5^-C_5_Me_5_)_2_Rh_2_(μ-dhnq)Cl_2_] (**1**)

5,8-Dihydroxy-1,4-naphthoquinone (dhnqH_2_) (30.8 mg, 0.162 mmol) was added to a solution of CH_3_COONa (27 mg, 0.324 mmol) in methanol (30 mL). The mixture was stirred for 1 h and [(η^5^-C_5_Me_5_)_2_Rh_2_(µ-Cl)_2_Cl_2_] (100 mg, 0.162 mmol) was added and stirred at room temperature. After stirring overnight the solvent was removed under reduced pressure and the solid was washed with water and dried under vacuum to give a yellow color compound. Yield 93 mg (78%). Calcd for C_30_H_34_Cl_2_O_4_Rh_2_: C, 49.00; H, 4.66. Found: C, 48.28; H, 4.36. IR (KBr pellets, cm^−1^): *ν* = 1532 s, 1418 m. ^1^H NMR (400 MHz, CD_2_Cl_2_): δ = 7.01 (s, 4H, dhnq), 1.60 (s, 30H, C_5_Me_5_) ppm. ^13^C{^1^H} NMR (100 MHz, CD_2_Cl_2_): δ = 8.63, 92.87, 112.75, 138.66 and 171.59 ppm.

### 3.3. Synthesis of [(η^5^-C_5_Me_5_)_2_Ir_2_(μ-dhnq)Cl_2_] (**2**)

5,8-Dihydroxy-1,4-naphthoquinone (dhnqH_2_) (24 mg, 0.125 mmol) was added to a solution of CH_3_COONa (20.6 mg, 0.250 mmol) in methanol (30 mL). The mixture was stirred for 1 h and [(η^5^-C_5_Me_5_)_2_Ir_2_(µ-Cl)_2_Cl_2_] (100 mg, 0.125 mmol) was added and stirred at room temperature. After stirring overnight the solvent was removed under reduced pressure and the solid was washed with water and dried under vacuum to give a grey color compound. Yield 79 mg (69%). Calcd for C_30_H_34_Cl_2_O_4_Ir_2_: C, 39.43; H, 3.75. Found: C, 38.72; H, 3.83. IR (KBr pellets, cm^−1^): *ν* = 1531 s, 1417 s. ^1^H NMR (400 MHz, CD_2_Cl_2_): δ = 7.09 (s, 4H, dhnq), 1.58 (s, 30H, C_5_Me_5_) ppm. ^13^C{^1^H} NMR (100 MHz, CD_2_Cl_2_): δ = 8.80, 84.74, 110.39, 138.48 and 168.21 ppm.

### 3.4. Synthesis of [(η^5^-C_5_Me_5_)_4_Rh_4_(μ-dhnq)_2_(μ-pyrazine)_2_](CF_3_SO_3_)_4_ {[**3**](CF_3_SO_3_)_4_}

A mixture of **1** (60 mg, 0.081 mmol) and AgCF_3_SO_3_ (42 mg, 0.163 mmol) in methanol (25 mL) was stirred at room temperature for 4 h and then filtered to remove the AgCl salt formed. Pyrazine (6.5 mg, 0.081 mmol) was added to the filtrate and the solution was stirred overnight at room temperature. The solvent was removed under reduced pressure and dichloromethane (3 mL) was added. Addition of diethyl ether to the dichloromethane solution precipitated the desired product as a green powder. The powder was removed by filtration and dried under vacuum. Yield: 41 mg (49%). Calcd for [C_68_H_76_N_4_O_8_Rh_4_] (CF_3_SO_3_)_4_: C, 41.47; H, 3.67; N, 2.69. Found: C, 41.47; H, 3.88; N, 2.64. IR (KBr pellets, cm^−1^): *ν* = 1535 s, 1418 m, 1271 s, 1158 m, 1031 s, 639 s. ^1^H NMR (400 MHz, CD_2_Cl_2_): δ = 8.81 (s, 8H, pyrazine), 7.20 (s, 8H, dhnq), 1.61 (s, 60H, C_5_Me_5_) ppm.^ 13^C{^1^H} NMR (100 MHz, CD_2_Cl_2_): δ = 8.34, 96.30, 111.65, 139.15, 148.96 and 171.61 ppm.

### 3.5. Synthesis of [(η^5^-C_5_Me_5_)_4_Ir_4_(μ-dhnq)_2_(μ-pyrazine)_2_](CF_3_SO_3_)_4_ {[**4**](CF_3_SO_3_)_4_}

A mixture of **2** (60 mg, 0.066 mmol) and AgCF_3_SO_3_ (34 mg, 0.132 mmol) in methanol (25 mL) was stirred at room temperature for 4 h and then filtered to remove the AgCl salt formed. Pyrazine (5.3 mg, 0.066 mmol) was added to the filtrate and the solution was stirred overnight at room temperature. The solvent was removed under reduced pressure and dichloromethane (3 mL) was added. Addition of diethyl ether to the dichloromethane solution gave the desired product as a grey powder. The powder was removed by filtration and dried under vacuum. Yield: 43 mg (54%). Calcd for [C_68_H_76_N_4_O_8_Ir_4_](CF_3_SO_3_)_4_: C, 35.41; H, 3.14; N, 2.29. Found: C, 36.21; H, 3.48; N, 2.33. IR (KBr pellets, cm^−1^): *ν* = 1533 s, 1427 s, 1275 s, 1155 s, 1030 s, 637 s. ^1^H NMR (400 MHz, CD_2_Cl_2_): δ = 8.88 (s, 8H, pyrazine), 7.38 (s, 8H, dhnq), 1.57 (s, 60H, C_5_Me_5_) ppm.^ 13^C{^1^H} NMR (100 MHz, CD_2_Cl_2_): δ = 8.28, 88.51, 113.75, 139.24, 149.28 and 168.71 ppm.

### 3.6. Synthesis of [(η^5^-C_5_Me_5_)_4_Rh_4_(μ-dhnq)_2_(μ-4,4′-bipyridine)_2_](CF_3_SO_3_)_4_ {[**5**](CF_3_SO_3_)_4_}

The metalla-rectangle **5** was obtained from **1** (60 mg, 0.081 mmol), AgCF_3_SO_3_ (42 mg, 0.163 mmol) and 4,4′-bipyridine (12.8 mg, 0.081 mmol) following the procedure described for [**3**](CF_3_SO_3_)_4_. Yield: 70 mg (77%). Calcd for [C_80_H_84_N_4_O_8_Rh_4_](CF_3_SO_3_)_4_: C, 45.09; H, 3.78; N, 2.50. Found: C, 44.77; H, 3.58; N, 2.34. IR (KBr pellets, cm^−1^): *ν* = 1535 s, 1414 m, 1271 s, 1157 m, 1031 s, 639 s. ^1^H NMR (400 MHz, CD_2_Cl_2_): δ = 8.36 (d, 8H, ^3^*J* = 8 Hz, bipyridine), 7.87 (d, 8H, ^3^*J =* 8 Hz, bipyridine), 7.08 (s, 8H, dhnq), 1.47 (s, 60H, C_5_Me_5_) ppm.^ 13^C{^1^H} NMR (100 MHz, CD_2_Cl_2_): δ = 8.42, 95.38, 111.94, 124.63, 139.16, 145.62, 151.68 and 171.58 ppm.

### 3.7. Synthesis of [(η^5^-C_5_Me_5_)_4_Ir_4_(μ-dhnq)_2_(μ-4,4′-bipyridine)_2_](CF_3_SO_3_)_4_ {[**6**](CF_3_SO_3_)_4_}

The metalla-rectangle **6** was obtained from **2** (60 mg, 0.066 mmol), AgCF_3_SO_3_ (34 mg, 0.132 mmol) and 4,4′-bipyridine (10.3 mg, 0.066 mmol) following the procedure described for [**4**](CF_3_SO_3_)_4_. Yield: 53 mg (72%). Calcd for [C_80_H_84_N_4_O_8_Ir_4_](CF_3_SO_3_)_4_·6 H_2_O: C, 37.33; H, 3.58; N, 2.07. Found: C, 36.79; H, 3.58; N, 2.82. IR (KBr pellets, cm^−1^): *ν* = 1533 s, 1427 m, 1276 s, 1156 m, 1031 s, 639 s. ^1^H NMR (400 MHz, CD_2_Cl_2_): δ = 8.47 (d, 8H, ^3^*J =* 8 Hz, bipyridine), 8.05 (d, 8H, ^3^*J =* 8 Hz, bipyridine), 7.31 (s, 8H, dhnq), 1.54 (s, 60H, C_5_Me_5_) ppm. ^13^C{^1^H} NMR (100 MHz, CD_2_Cl_2_): δ = 8.45, 87.41, 13.92, 124.99, 139.18, 145.18, 151.34 and 168.54 ppm.

### 3.8. Synthesis of [(η^5^-C_5_Me_5_)_4_Rh_4_(μ-dhnq)_2_(μ-1,2-bis(4-pyridyl)ethane)_2_](CF_3_SO_3_)_4_ {[**7**](CF_3_SO_3_)_4_}

The metalla-rectangle **7** was obtained from **1** (60 mg, 0.081 mmol), AgCF_3_SO_3_ (42 mg, 0.163 mmol) and 1,2-bis(4-pyridyl)ethylene (14.9 mg, 0.081 mmol) following the procedure described for [**3**](CF_3_SO_3_)_4_. Yield: 75 mg (81%). Calcd for [C_84_H_88_N_4_O_8_Rh_4_](CF_3_SO_3_)_4_: C, 46.16; H, 3.87; N, 2.45. Found: C, 45.66; H, 3.53; N, 2.45. IR (KBr pellets, cm^−1^): *ν* = 1535 s, 1417 m, 1271 s, 1160 m, 1032 s, 639 s. ^1^H NMR (400 MHz, CD_2_Cl_2_): δ = 8.27 (d, 8H, ^3^*J =* 8 Hz, pyridyl), 7.70 (d, 8H, ^3^*J =* 8 Hz, pyridyl), 7.42 (s, 4H, CH=CH), 7.16 (s, 8H, dhnq), 1.57 (s, 60H, C_5_Me_5_) ppm. ^13^C{^1^H} NMR (100 MHz, CD_2_Cl_2_): δ = 8.44, 95.26, 111.86, 124.93, 131.77, 139.10, 146.68, 150.71 and 171.50 ppm.

### 3.9. Synthesis of [(η^5^-C_5_Me_5_)_4_Ir_4_(μ-dhnq)_2_(μ-1,2-bis(4-pyridyl)ethane)_2_](CF_3_SO_3_)_4_ {[**8**](CF_3_SO_3_)_4_}

The metalla-rectangle **8** was obtained from **2** (60 mg, 0.066 mmol), AgCF_3_SO_3_ (34 mg, 0.132 mmol) and 1,2-bis(4-pyridyl)ethylene (12 mg, 0.066 mmol) following the procedure described for [**4**](CF_3_SO_3_)_4_. Yield: 60 mg (69%). Calcd for [C_84_H_88_N_4_O_8_Ir_4_](CF_3_SO_3_)_4_·6 H_2_O: C, 38.37; H, 3.66; N, 2.03. Found: C, 38.02; H, 3.58; N, 2.12. IR (KBr pellets, cm^−1^): *ν* = 1532 s, 1427 m, 1275 s, 1157 m, 1031 s, 639 s. ^1^H NMR (400 MHz, CD_2_Cl_2_): δ = 8.28 (d, 8H, ^3^*J =* 8 Hz, pyridyl), 7.78 (d, 8H, ^3^*J =* 8 Hz, pyridyl), 7.49 (s, 4H, CH=CH), 7.29 (s, 8H, dhnq), 1.53 (s, 60H, C_5_Me_5_) ppm.^ 13^C{^1^H} NMR (100 MHz, CD_2_Cl_2_): δ = 8.48, 87.19, 113.86, 125.30, 131.89, 139.10, 147.08, 150.26 and 168.41 ppm.

### 3.10. Cell Culture and Inhibition of Cell Growth

Human A2780 and A2780cisR ovarian carcinoma cells and HEK293 cells were obtained from the European Collection of Cell Cultures (ECACC) (Salisbury, UK). Cells were cultured in either RPMI-1640 with GlutaMAX (A2780, A2780cisR) or DMEM (Dulbecco’s Modified Eagle Medium) high glucose with GlutaMAX (HEK 293) medium containing 10% fetal bovine serum (FBS) and penicillin at 37 °C and 5% CO_2_. Cytotoxicity was determined using the MTT (3-(4,5-dimethylthiazol-2-yl)-2,5-diphenyltetrazolium bromide) assay (see below). Cells were seeded in 96 well plates by the addition of cells as a suspension in their respective media containing 10% FBS (100 μL per well, approximately 4300 cells) and pre-incubated for 24 h.

Fresh stock solutions of the compounds were prepared in DMSO just before injections, then the stock solution were diluted by addition to the culture medium [RPMI (Roswell Park Memorial Institute medium) or DMEM for A2780 and A2780cisR or HEK 293, respectively]. The stock solutions were serially diluted to give compound solutions of the desired concentrations. Complex solutions (100 μL) were then added to plate wells (yielding final compound solutions in the range 0 to 5 μM) and the plates incubated for a further 72 h.

Subsequently, MTT (3-(4,5-dimethyl-2-thiazolyl)-2,5-diphenyl-2H-tetrazolium bromide) solution (20 μL, 5 mg/mL in H_2_O) was added to each well and the plates incubated for a further 2 h. The culture medium was then aspirated and the violet formazan precipitate produced by mitochondrial dehydrogenases of living cells was dissolved by the addition of DMSO (100 μL) to each well. The absorbance of the resultant solutions at 590 nm, which is directly proportional to the number of surviving cells, was recorded using a multiwell plate reader. The percentage of surviving cells was determined by measurement of the absorbance of wells corresponding to untreated control cells. The reported IC_50_ values are based on the mean values from two independent experiments; each concentration level per experiment was evaluated in triplicate, and those values are reported in Table 3.

### 3.11. Single-Crystal X-ray Structure Analysis

Crystals of compounds [**3**](CF_3_SO_3_)_4_, [**5**](CF_3_SO_3_)_4_ and [**6**](CF_3_SO_3_)_4_ were mounted on a Stoe Image Plate Diffraction system equipped with a ϕ circle goniometer, using Mo-Kα graphite monochromatic radiation (λ = 0.71073 Å) with ϕ range 0–200°. The structures were solved by direct methods using the program SHELXS-97, while the refinement and all further calculations were carried out using SHELXL-97 [40]. The H-atoms were included in calculated positions and treated as riding atoms using the SHELXL default parameters. The non-H atoms were refined anisotropically, using weighted full-matrix least-square on *F*^2^. In **6**, the solvent molecules were highly disordered and a data set corresponding to omission of the missing solvent was generated using the SQUEEZE algorithm [41] and the structure was refined to convergence. These missing solvent molecules are probably dichloromethane molecules, which fit perfectly with the size of the voids, the electron counts and the crystal packing of compound [**5**](CF_3_SO_3_)_4_, which possesses two molecules of dichloromethane per asymmetric unit. Crystallographic details for [**3**](CF_3_SO_3_)_4_, [**5**](CF_3_SO_3_)_4_ and [**6**](CF_3_SO_3_)_4_ are summarized in Table 2. Figure 3, Figure 4 and Figure 5 were drawn with ORTEP [42] and Figure 6 was drawn with Mercury [43].

CCDC-959397 [**3**](CF_3_SO_3_)_4_·4CH_2_Cl_2_, 959398 [**5**](CF_3_SO_3_)_4_·2CH_2_Cl_2_ and 959399 [**6**](CF_3_SO_3_)_4_·solvent contain the supplementary crystallographic data for this paper. These data can be obtained free of charge at www.ccdc.cam.ac.uk/conts/retrieving.html [or from the Cambridge Crystallographic Data Centre, 12, Union Road, Cambridge CB2 1EZ, UK; fax: (internat.) +44-1223/336-033; E-mail: deposit@ccdc.cam.ac.uk].

## 4. Conclusions

The antiproliferative activities of a series of half-sandwich Rh(III) and Ir(III) tetranuclear metalla-cycles have been evaluated* in vitro* against the human ovarian A2780 (cisplatin sensitive) and A2780cisR (cisplatin resistant) cancer cell lines and on non-tumorigenic human embryonic kidney HEK293 cells. These metalla-rectangles have been found to be highly cytotoxic with IC_50_ in the low micromolar range. The cationic charge, the size, the nature of the metal ion, and the presence of a hydrophobic cavity in these metalla-materials, are all plausible factors which can contribute to their high biological activity. These results further confirm the great potential of metalla-materials in the field of biology [1,2,3,4,5,6].
